# Selenate and selenite transporters in proso millet: Genome extensive detection and expression studies under salt stress and selenium

**DOI:** 10.3389/fpls.2022.1060154

**Published:** 2022-11-30

**Authors:** Naveed Ul Mushtaq, Khalid M. Alghamdi, Seerat Saleem, Faamiya Shajar, Inayatullah Tahir, Ahmad Bahieldin, Reiaz Ul Rehman, Khalid Rehman Hakeem

**Affiliations:** ^1^ Department of Bioresources, School of Biological Sciences, University of Kashmir, Srinagar, India; ^2^ Department of Biological Sciences, Faculty of Science, King Abdulaziz University, Jeddah, Saudi Arabia; ^3^ Department of Botany, School of Biological Sciences, University of Kashmir, Srinagar, India; ^4^ Princess Dr. Najla Bint Saud Al-Saud Center for Excellence Research in Biotechnology, King Abdulaziz University, Jeddah, Saudi Arabia; ^5^ Department of Public Health, Daffodil International University, Dhaka, Bangladesh

**Keywords:** millet, transporters, selenite, protein-protein interaction, protein modeling

## Abstract

Crops are susceptible to a variety of stresses and amongst them salinity of soil is a global agronomic challenge that has a detrimental influence on crop yields, thus posing a severe danger to our food security. Therefore, it becomes imperative to examine how plants respond to salt stress, develop a tolerance that allows them to live through higher salt concentrations and choose species that can endure salt stress. From the perspective of food, security millets can be substituted to avoid hardships because of their efficiency in dealing with salt stress. Besides, this problem can also be tackled by using beneficial exogenous elements. Selenium (Se) which exists as selenate or selenite is one such cardinal element that has been reported to alleviate salt stress. The present study aimed for identification of selenate and selenite transporters in proso millet (*Panicum miliaceum* L.), their expression under NaCl (salt stress) and Na_2_SeO_3_ (sodium selenite)treatments. This study identified eight transporters (RLM65282.1, RLN42222.1, RLN18407.1, RLM74477.1, RLN41904.1, RLN17428.1, RLN17268.1, RLM65753.1) that have a potential role in Se uptake in proso millet. We analyzed physicochemical properties, conserved structures, sub-cellular locations, chromosome location, molecular phylogenetic analysis, promoter regions prediction, protein-protein interactions, three-dimensional structure modeling and evaluation of these transporters. The analysis revealed the chromosome location and the number of amino acids present in these transporters as RLM65282.1 (16/646); RLN42222.1 (1/543); RLN18407.1 (2/483); RLM74477.1 (15/474); RLN41904.1 (1/521); RLN17428.1 (2/522); RLN17268.1(2/537);RLM65753.1 (16/539). The sub-cellular locations revealed that all the selenite transporters are located in plasma membrane whereas among selenate transporters RLM65282.1 and RLM74477.1 are located in mitochondria and RLN42222.1 and RLN18407.1 in chloroplast. The transcriptomic studies revealed that NaCl stress decreased the expression of both selenate and selenite transporters in proso millet and the applications of exogenous 1µM Se (Na_2_SeO_3_) increased the expression of these Se transporter genes. It was also revealed that selenate shows similar behavior as sulfate, while selenite transport resembles phosphate. Thus, it can be concluded that phosphate and sulphate transporters in millets are responsible for Se uptake.

## Introduction

Despite rising agricultural expenses and climate changes, millets guarantee food security and nourishment. In addition to being healthy, they have added well-being benefits and require significantly fewer efforts to grow (Bandyopadhyay et al., 2017). Amidst the mounting concern regarding climate change, these characteristics suggest millets as plants of preference for humankind. A minor pseudocereal millet crop, proso millet (*Panicum miliaceum* L.) has been valued as a healthy food for centuries. About ten thousand years ago, it originated in Northern China as a prime domesticated plant of the Poaceae family (Li et al., 2021). Crops are susceptible to a variety of stresses like toxic metals, fluctuating temperatures, pesticides, salinity as well as biotic stresses and millets are no exception to these stresses (Li et al., 2021; Mushtaq et al., 2021). Proso millet has the advantage of being a skillful propagator in poor environmental conditions, places where other crops have meagre chances of growth (Nazir et al., 2021). Soil salinity is a global agronomic challenge that has a detrimental influence on crop yields and thus threatens food security (Ibrahimova et al., 2021). Sodium and chloride ions accumulate in the tissues of plants causing ionic and osmotic stress (Zhao et al., 2021). In the event of ionic stress, potassium and sodium homeostasis is disrupted, inhibiting plant growth. Higher levels of sodium impede the enzymatic function and destabilize protein structure by messing with the surface charges (Singh and Roychoudhury, 2021). On the other hand osmotic stress results in more reactive oxygen species (ROS) generation in many compartments, causing oxidative damage, reduction in photosynthesis, stomatal limitations, lipid peroxidation, protein and DNA damage and reduced enzyme activity (Isayenkov and Maathuis, 2019; Ahmad et al., 2021). In this context as salinity is posing a severe danger to our food security, we must examine how the response of plants towards salt and thus identify and produce salt-tolerant plants (Ibrahimova et al., 2021). It is critical to envisage plant enhancements that allow them to live in high salt concentrations, as well as to choose plant species that can withstand salt stress. This could be achieved by using efficient crop varieties like millets and by the application of beneficial exogenous compounds. Selenium (Se) is an essential compound that has been shown to alleviate salt stress. At relatively lower concentrations of Se, plant growth and development can be improved (Hasanuzzaman et al., 2020). Se can increase the activity of antioxidant enzymes (Mushtaq et al., 2021), enhancing tolerance towards drought (Ghouri et al., 2021), salt stress (Shah et al., 2020; Taha et al., 2021), low temperature (Liu et al., 2021), toxic metals (Zhou et al., 2021), and ultraviolent light-induced stresses (Mata-Ramírez et al., 2019). Se was discovered by Swedish chemist Jacob Berzelius (1817) and the word is derived from the ancient Greek word, “Selene” meaning moon (Monesh Babu and Preetha, 2021). In the periodic table, Se is a member of group 16, and due to this reason, it has similar chemical characteristics as Sulfur (S) (Bodnar et al., 2012). In the earth’s lithosphere, Se exists in different amounts (Floor and Román-Ross, 2012) and its content and form depend on the soil type, its organic matter, the amount of rainfall and topography (Zhu et al., 2009; Gupta and Gupta, 2017). Se is abundant in soils formed by igneous rock, granite, sandstone, limestone and generally scarce in soils formed by temperate and humid climates. In mineral-enriched soils, Se levels vary by 14 mg/kg regardless of soil depth (Hossain et al., 2021). The inorganic forms of Se consist of selenate, selenite, selenide and elemental Se (Bodnar et al., 2012) while as organic forms include selenocysteine (Se-Cys) and selenomethionine (SeMet) (Wu et al., 2015; Ahmad et al., 2021). Se can cause toxicity in humans at higher concentrations, even though it is a beneficial micronutrient. However, it is still being debated whether Se is essential for plants or not, but, it has shown protective roles in several plants when applied at lower concentrations (Gupta and Gupta, 2017; Lanza and Dos Reis, 2021). Furthermore, studies report Se as a beneficial element that acts as an antioxidant, anti-senescent and provides oxidative stress tolerance to plants (Ahmad et al., 2021).

Most plant species utilize only a little amount of Se from soil and they differ in their ability to accumulate Se in their tissues (White, 2016). Se absorption, transport, and translocation are influenced by a variety of parameters, including the quantity and type of Se in the environment, plant species, medium pH, and the presence of Sulphur and phosphorus (Rasool et al., 2022). Agricultural soils are predominantly richer in selenate than selenite and the selenate being more water soluble is readily absorbed as compared to selenite (Raina et al., 2021). As a result of the chemical analogy of selanate/selenite with sulphate and phosphate, their behavior in metabolism and transport in plants is closely related (Raina et al., 2021). A high concentration of Se seems to promote the pathway of “Starch and sucrose metabolism” as its treatment resulted in the accumulation of both forms of carbohydrates in potato (*Solanum tuberosum* L.) (Turakainen et al., 2004). We speculate that carbohydrate-active enzymes (CAZymes) of class glycoside hydrolases (GH) that are mostly enriched in this pathway can be the target of Selenium. The co-transport of selenate and selenite occurs with protons (Smith et al., 2000) and thus their transport occurs with the help of sulphate and phosphate transporters (Feist and Parker, 2001; White et al., 2004). In *Arabidopsis thaliana* Q9LIK9, a sulfur transporter controls the reduction of selenate and assists selenate and sulfur absorption and assimilation (Pilon-Smits et al., 1999). In rice, a phosphate transporter (OsPT2) is responsible for the active uptake of selenite (Zhang et al., 2014). Similar reports on the role of phosphate transporter in selenite uptake have been revealed in many studies (Li et al., 2008; Gu et al., 2016). The mechanism of selenium uptake in proso millet is not well understood and the present study aims to identify selenate and selenite transporters and their expression under salt stress and Se (Na_2_SeO_3_) application.

## Material and methods

### Sequence retrieval

For this study, the experimentally reviewed selenate transporter amino acid sequence of *Arabidopsis thaliana* L. Q9LIK9 (APS1_ARATH) and selenite transporter sequence of *Oryza sativa* L. Q8GSD9 (PTH1-2 PT1, PT2, Os03g0150800) were retrieved from UniProtKB database (https://www.uniprot.org/). In *Arabidopsis thaliana* Q9LIK9, a sulfur transporter controls the reduction of selenate and assists selenate and sulfur absorption and assimilation (Pilon-Smits et al., 1999). Q8GSD9, is a phosphate transporter, that is involved in the active uptake of selenite in rice (Zhang et al., 2014; Gu et al., 2016). These proteins were used as a source for a local pBLAST search against the *Panicum miliaceum* L. (Proso millet) database. The NCBI protein blast tool (https://blast.ncbi.nlm.nih.gov/Blast.cgi) was employed to search for homologous genes in Proso millet.

### Physiochemical, domains and transmembrane helices analysis

The physicochemical properties of resultant transporters were calculated with the help of ProtParam (https://web.expasy.org/protparam). The trans-membrane domains were identified by the TMHMM server v2 (Krogh et al., 2001). Pfam 35.0 database was used for domain detection (http://pfam.xfam.org).

### Conserved structure prediction, chromosome and sub-cellular location detection

The conserved structures of the screened selenite and selenate proteins were characterized through the CDD-Search in NCBI and TBtools. Among the result, we predicted the sub-cellular locations of proteins using CELLO V2.5 as per (Yu et al., 2006). The chromosomal locations of the results were performed by an integrative toolkit TBtools and by phenogram (http://visualization.ritchielab.org/phenograms/plot) (Chen et al., 2020).

### Evolutionary analysis

The molecular phylogenetic analysis of selenite and selenate transporter proteins was performed using MEGAX v 11 (Akbudak and Filiz, 2020; Tamura et al., 2021). The evolutionary history was inferred by using the Maximum Likelihood method and Poisson correction model (Zuckerkandl and Pauling, 1965). Initially, the selenate sequence of Q9LIK9 and selenite transporter sequence

Q8GSD9 were used for the protein-protein blast in NCBI (https://blast.ncbi.nlm.nih.gov/Blast.cgi). These respective sequences were searched with similar proteins in their genome (i.e. Q9LIK9 against *Arabidopsis thaliana* and Q8GSD9 against *Oryza sativa* L.). Among the results 32 amino acid sequences for selenate and 52 for selenite were used with selected Se (both selenate and selenite) transporters in proso millet to construct a tree. Clustal W was used for aligning the sequences. Gap opening penalty of 10 and gap extension penalty of 0.1 in pair wise alignment and opening penalty of 10 and extension penalty of 0.2 in multiple alignments was kept with delay divergent cut-off as 30%. Tajima’s relative rate test with complete deletion method was carried to check P-values (Tajima, 1993). The phylogeny construction was performed by Maximum Likelihood method, Poisson model and by applying Neighbor-Joining and BioNJ algorithms. The bootstrap consensus tree was inferred from 500 replicates.

### Promoter region prediction

The gene sequences of RLM65282.1, RLN42222.1, RLN18407.1, RLM74477.1, RLN41904.1, RLN17428.1, RLN17268.1, RLM65753.1 were screened for promoter regions. The gene sequences were converted into nucleotide sequences by Sequence manipulation suite reverse translate (https://www.bioinformatics.org/sms2/rev_trans.html). These nucleotides were used to predict location of Pol II promoter in regions (Knudsen, 1999) (https://services.healthtech.dtu.dk/).

### Protein-protein interaction analysis

The protein-protein interactions (PPI) of the selected transporter proteins were compared and examined by STRING V 11.5 and the multiple sequence searches of these transporter proteins were performed with proso millet.

### Three-dimensional structure modeling and model assessment

The three-dimensional structures of the transporter proteins from proso millet were determined using Swiss modeling software (https://swissmodel.expasy.org). For the target sequence of interest, a Swiss repository was searched (https://swissmodel.expasy.org/repository). The target sequence and sequences with known structures comparable to the query sequence were aligned using homology modeling. A structural model of the target is created from the sequence alignment and template structure. Based on the obtained BLAST results, the sequence with the highest sequence identity score was chosen. To evaluate Swiss models, the in-build structure assessment tool was employed (https://swissmodel.expasy.org/assess).

### Plant growth and treatments

The seeds were sterilized in 70% (v/v) ethanol for 1 minute before being rinsed with sterile distilled water. Seeds were surface sterilized for 3 minutes in 0.1 percent HgCl_2_ (Merck, India) solution (w/v), followed by washing in sterilized distilled water. The seeds were sown in pots that measured 20 cm in diameter that contained autoclaved sand. A controlled environment with a 26 ± 1°C temperature and a 16-h photoperiod was maintained (Hussain et al., 2008; Desoky et al., 2019). Three sets of plants were grown with three replicates each with treatments given after 14 days of sowing. The concentrations of NaCl (150mM) and Se (1 μM Na_2_SeO_3_) were applied to the pots (Shah et al., 2020; Rasool et al., 2020). A Hoagland nutrient medium (pH 6.5) containing all macro and micronutrients was used to grow the seedlings and harvesting for analysis was done 24 days after sowing.

### Transcriptomic studies of identified transporters genes

The samples were prepared using liquid nitrogen and the trizol reagent (Invitrogen) was used to isolate total RNA from plant material (Deepa et al., 2014). Genomic DNA contamination was removed by adding RNase-free DNase (Promega) and to evaluate RNA quality Agilent 2100 Bioanalyzer (Agilent Technologies) was used. To validate and study the expression of transporter genes, cDNA was synthesized using the revert aid cDNA synthesis kit (Thermo Scientific) following the manufacturer’s protocol. The gene expression was carried out for the control plant, 150 mM of NaCl treated and 150 mM of NaCl + 1 μM Se treated plants.

### Data analysis

XLSTAT 2021 and graph pad prism v6 was used for data analysis.

## Results

### Sequence retrieval, physiochemical, domains and transmembrane helices analysis

**Figure 1:**
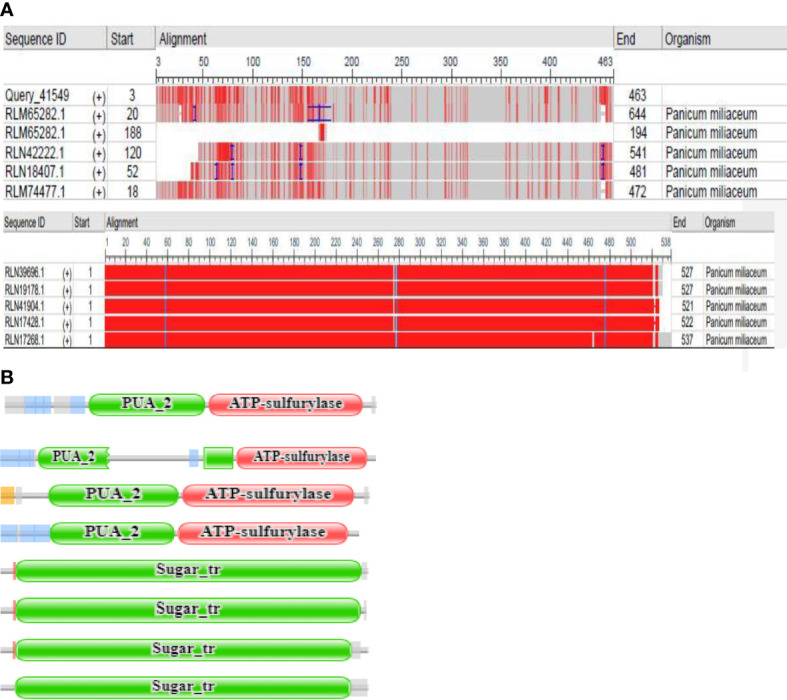
**(A)** Multiple sequence view of query sequence and results **(B)** Pfam domains of RLM65282.1, RLN42222.1, RLN18407.1 and RLM74477.1, RLN41904.1, RLN17428.1,RLN17268.1, RLM65753.1 respectively.

**Figure 2:**
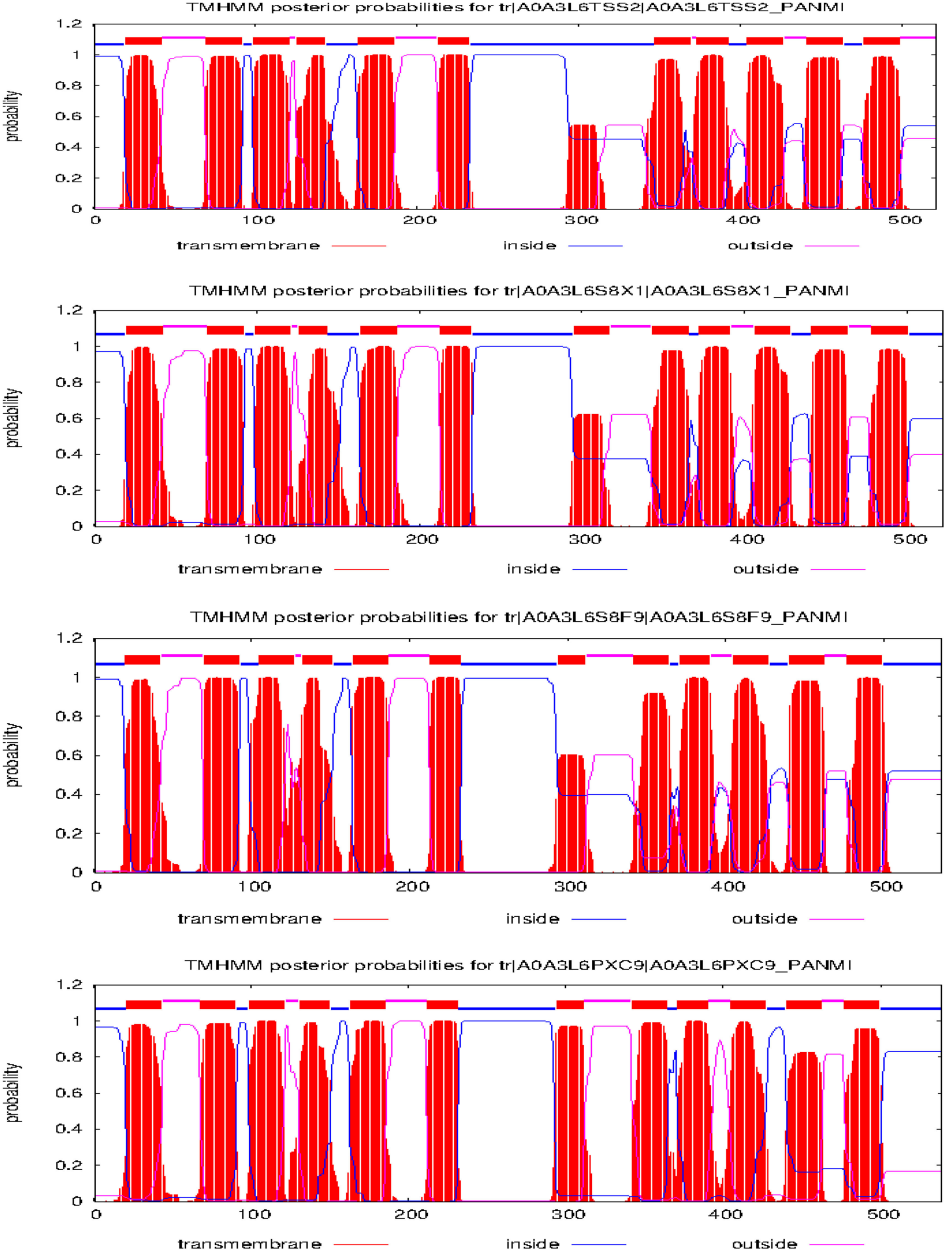
Trans-membrane helices prediction of selenite transporters in proso millet.

From the NCBI results, a total of 8 sequences from *Panicum miliacieum* L. produced significant alignments. Four sequences produced alignments for selenate transporters in *Arabidopsis thaliana* L. and four for selenite transporters in *Oryza sativa* L **Table 1**. Among selenate transporters, the hypothetical protein (RLM65282.1) produced 99% query cover, two ATP sulfurylase 3 proteins and one ATP sulfurylase 2-like protein produced 89%, 91% and 99% query cover respectively. Among selenite transporters the hypothetical protein (RLN41904.1), Inorganic phosphate transporter 1-1, putative inorganic transporter 1-12 and putative inorganic transporter 1-5 produced query cover of 98%, 98%, 99% and 98% respectively. From these results, it is evident that sulphate transporters may be responsible for selenate uptake and phosphate transporters for selenite uptake. The physicochemical properties, trans-membrane domains, sub-cellular locations, theoretical isoelectric point, instability index, aliphatic index, number of amino acids and molecular weight of selenate and selenite transporters are given in **Tables 2**, **3**. While comparing the total number of charged residues in the case of selenite transporters, it is evident that RLM65282.1 is neutral because it contains an equal number of positive and negative charged residues (Khan et al., 2015), while all others i.e. RLN42222.1, RLN18407.1 and RLM74477.1 are positively charged. However, no transmembrane helices were found in these selenate transporters. While comparing the total number of charged residues in selenite transporters, it was found that all of them contained dominant positive charged residues and thus the net charge of all these is positive. RLN41904.1 contained 11 transmembrane helices whereas RLN17428.1, RLN17268.1 and RLM65753.1 contained 12 transmembrane helices in each. The Multiple sequence alignment and Pfam domains of selenate and selenite transporters are shown in **Figure 1** and the transmembrane helices for four selenite transporters only are shown in **Figure 2**.

**Table 1:** Percentage identity of selenate and selenite transporter sequence from *Panicum miliaceum* L. with *Arabidopsis thaliana* L. and *Oryza sativa* L.

S.No.	Selenate Transporter sequences	Max. score	Total score	Query cover	Percent identity	Length	Accession
1	hypothetical protein C2845_PM16G00810	525	678	99%	85.14%	646	RLM65282.1
2	ATP-sulfurylase 3	665	665	89%	76.3%	543	RLN42222.1
3	ATP-sulfurylase 3	662	662	91%	76.05%	483	RLN18407.1
4	ATP sulfurylase 2-like	666	666	99%	73.32%	474	RLM74477.1
5	Hypothetical protein C2845_PM01G41610	922	922	98%	85.41%	521	RLN41904.1
6	Inorganic phosphate transporter 1-1	897	897	98%	85.22%	522	RLN17428.1
7	Putative inorganic phosphate transporter 1-12	722	722	99%	72.95%	537	RLN17268.1
8	Putative inorganic phosphate transporter 1-5	784	784	98%	71.37%	539	RLM65753.1

**Table 2:** Predicted physiochemical properties of selenate transporters.

Parameters	Values for RLM65282.1	Values for RLN42222.1	Values for RLN18407.1	Values for RLM74477.1
Number of amino acids	646	543	483	474
Molecular weight of the molecule	72171.69 Da	59822.43 Da	53252.88 Da	52448.19 Da
Theoretical isoelectric point (pI)	7.4	9.82	8.85	8.38
The total number of negatively charged residues (Asp + Glu)	77	61	60	54
The total number of positively charged residues (Arg + Lys)	77	78	64	56
Molecular Formula	C_3235_H_5075_N_927_O_913_S_19_	C_2639_H_4210_N7_98_O_758_S_18_	C_2366_H_3736_N_692_O_679_S_16_	C_2348_H_3688_N_674_O_661_S_16_
Total no. of atoms	10169	8423	7489	7387
Extinction coefficient M^-1^ cm^-1^, at 280 nm measured in water (assuming all pairs of Cys residues form cystines)	102120	60515	60515	56505
Half-life estimate	30 hours (Mammals >20 hours (Yeast). >10 hours (*E.coli*)	30 hours (Mammals >20 hours (Yeast). >10 hours (*E.coli*)	30 hours (Mammals >20 hours (Yeast). >10 hours (*E.coli*)	30 hours (Mammals >20 hours (Yeast). >10 hours (*E.coli*)
Instability index	50.71 (Unstable)	56.72 (Unstable)	51.57 (Unstable)	58.61 (Unstable)
Aliphatic index	89.85	80.42	83.71	86.69
Grand average of hydropathicity	-0.302	-0.419	-0.317	-0.314
Sub-cellular location	Mitochondrial (Predicted)	Chloroplastic	Chloroplastic	Mitochondrial(Predicted)
Trans membrane helices	0/outside	0/outside	0/outside	0/outside

**Table 3:** Predicted physiochemical properties of selenite transporters.

Parameters	Values for RLN41904.1	Values for RLN17428.1	Values for RLN17268.1	Values for RLM65753.1
Number of amino acids	521	522	537	539
Molecular weight of the molecule	56766.91 Da	57085.31 Da	58405.89 Da	59138.29 Da
Theoretical isoelectric point (pI)	8.11	8.87	8.91	8.32
The total number of negatively charged residues (Asp + Glu)	35	34	34	40
The total number of positively charged residues (Arg + Lys)	37	40	41	43
Molecular Formula	C_2627_H_3979_N_657_O_702_S_24_	C_2642_H_4013_N_669_O_702_S_22_	C_2695_H_4114_N_682_O_723_S_24_	C_2721_H_4106_N6_84_O_742_S_27_
Total no. of atoms	7989	8048	8238	8280
Extinction coefficient M^-1^ cm^-1^, at 280 nm measured in water (assuming all pairs of Cys residues form cystines)	83685	90675	79675	79675
Half-life estimate	30 hours (Mammals >20 hours (Yeast) >10 hours (E. coli)	30 hours (Mammals >20 hours (Yeast) >10 hours (E. coli)	30 hours (Mammals >20 hours (Yeast) >10 hours (E. coli)	30 hours (Mammals >20 hours (Yeast) >10 hours (E. coli)
Instability index	30.96 (stable)	30.97 (stable)	34.57 (stable)	35.24 (stable)
Aliphatic index	91.29	92.59	92.03	81.17
Grand average of hydropathicity	0.389	0.387	0.394	0.218
Sub-cellular location	Plasma Membrane	Plasma Membrane	Plasma Membrane	Plasma Membrane
Trans membrane helices	11	12	12	12

### Conserved structure prediction, chromosome and sub-cellular location detection

**Figure 3:**
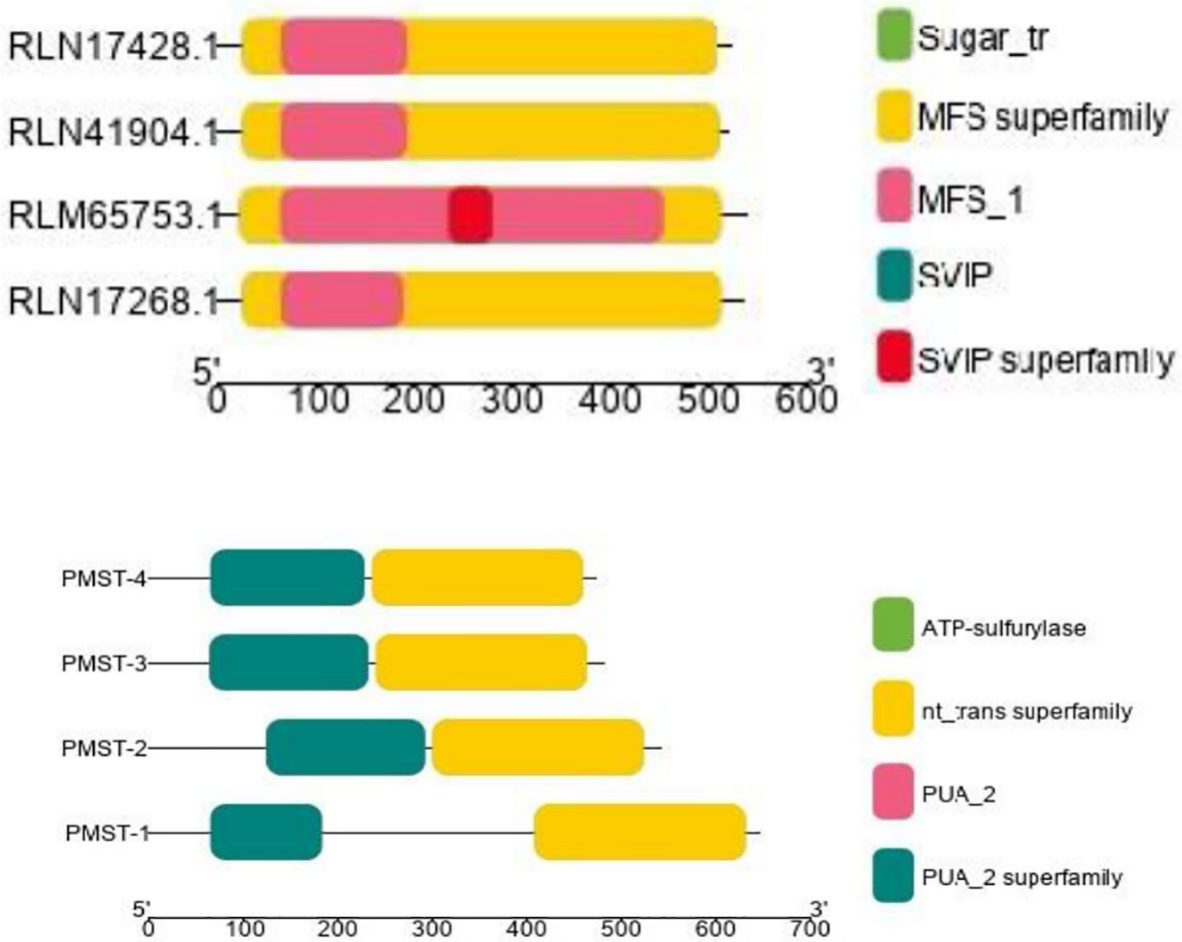
CDD of Selenite and selenate transporters in Proso millet respectively. PMST 1-4 represents RLM65282.1, RLN42222.1, RLN18407.1 and RLM74477.1 and PMPT 1-4 represents RLN41904.1, RLN17428.1, RLN17268.1 and RLM65753.1.

**Figure 4:**
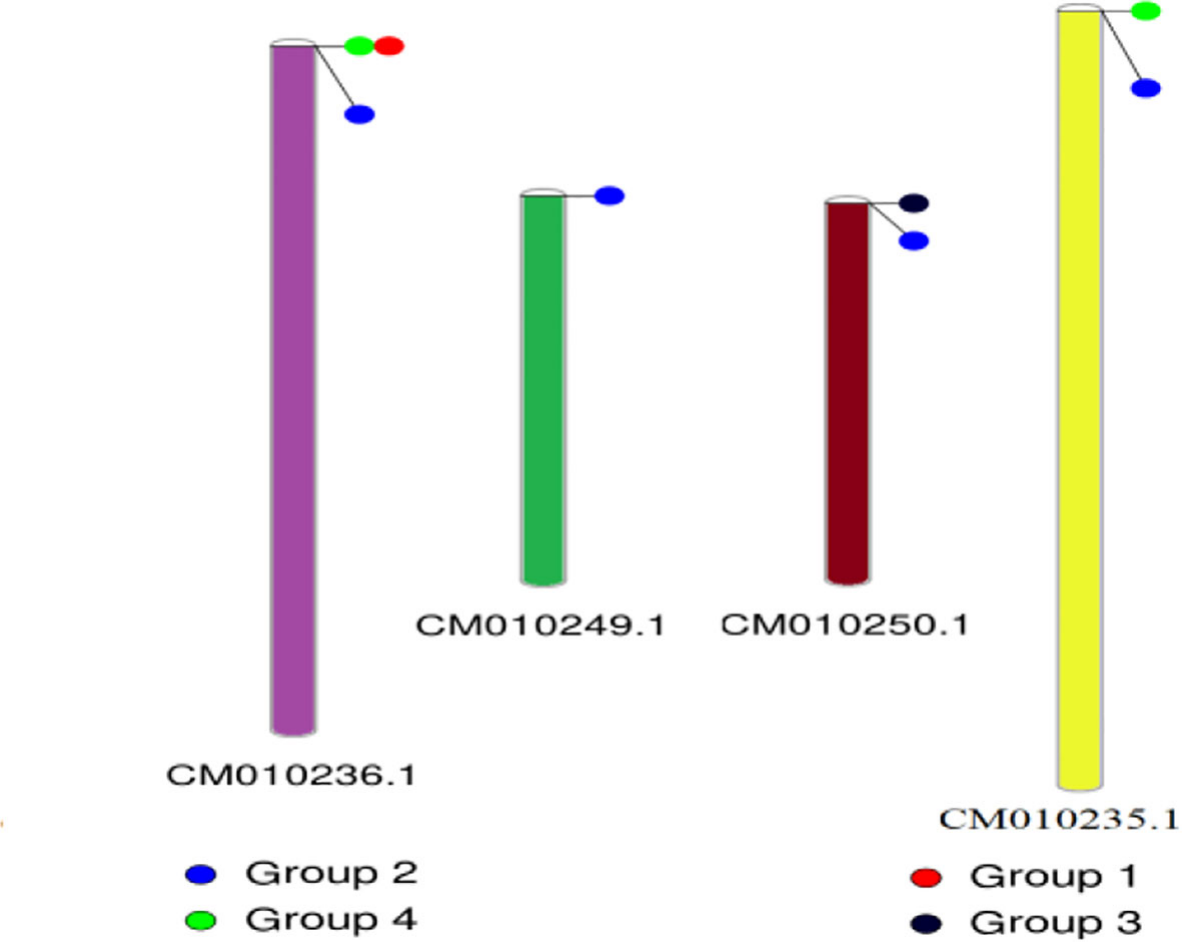
Chromosomal location of different transporters. CM010236.1, CM010249.1, CM010250.1 and CM010235.1 represents chromosome number 2, 15, 16 and 1 respectively.

The conserved structures of the screened selenite and selenate transporters are shown in **Figure 3**, and the chromosomal location and the location of the transporter genes are given in **Figure 4** and **Table 4**. Among selenate transporters, RLM65282.1, RLN42222.1, RLN18407.1 and RLM74477.1 are located on chromosome numbers 16, 1, 2 and 15 respectively, whereas selenite transporters RLN41904.1, RLN17428.1, RLN17268.1 and RLM65753.1 are located on chromosome numbers 1,2,2 and 16 respectively. The predicted sub-cellular locations of all the selenate transporters indicate that RLM65282.1 and RLM74477.1 are located in mitochondria whereas RLN42222.1, RLN18407.1 in the chloroplast. The sub-cellular locations of all the selenite transporters indicate that they may be located in plasma membrane **Tables 2**, **3**.

**Table 4:** Chromosomal studies of transporters.

Genes	Location on chromosome	Start position	Group based on tree
RLM65282.1	16	68	Group 2
RLN42222.1	1	127	Group 2
RLN18407.1	2	67	Group 2
RLM74477.1	15	68	Group 2
RLN41904.1	1	12	Group 4
RLN17428.1	2	8	Group 4
RLN17268.1	2	8	Group 1
RLM65753.1	16	10	Group 3

### Evolutionary analysis and promoter region prediction

**Figure 5:**
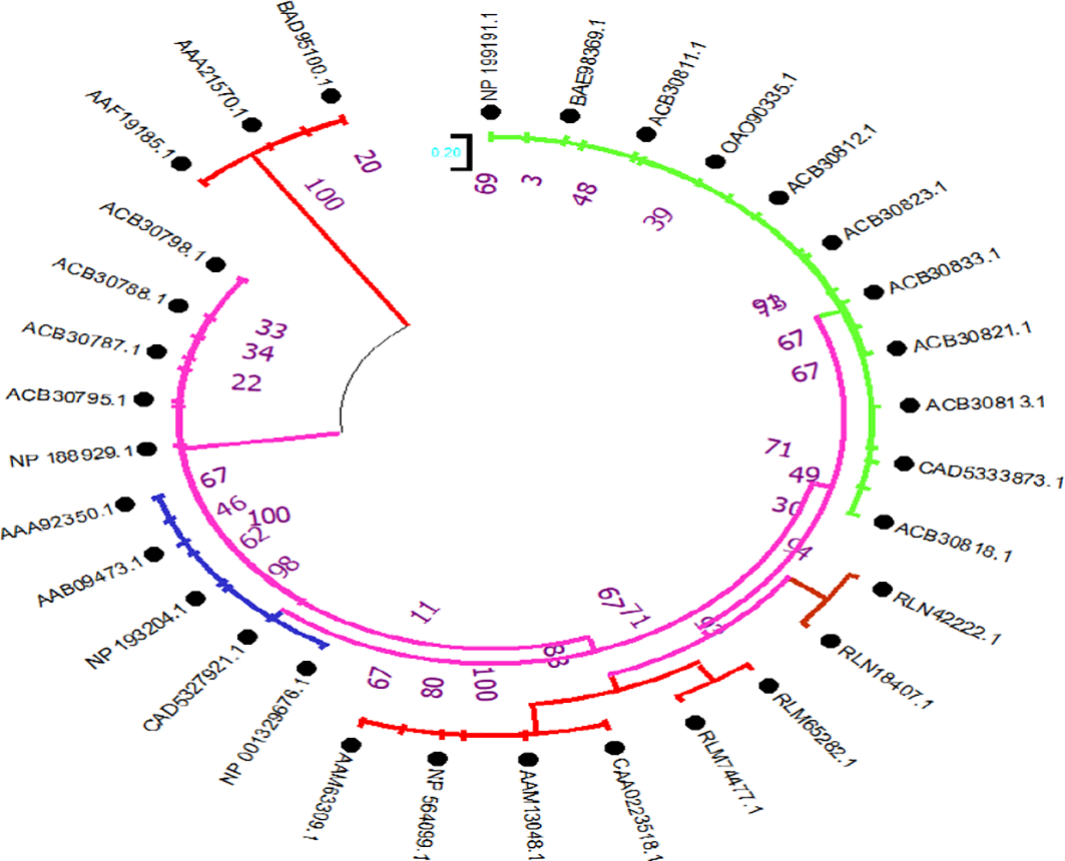
Phylogenetic analysis of selenate transporters among *A. thaliana* selenate transporters. The trees were constructed by the maximum likelihood method with 500 bootstrap replicates.

**Figure 6:**
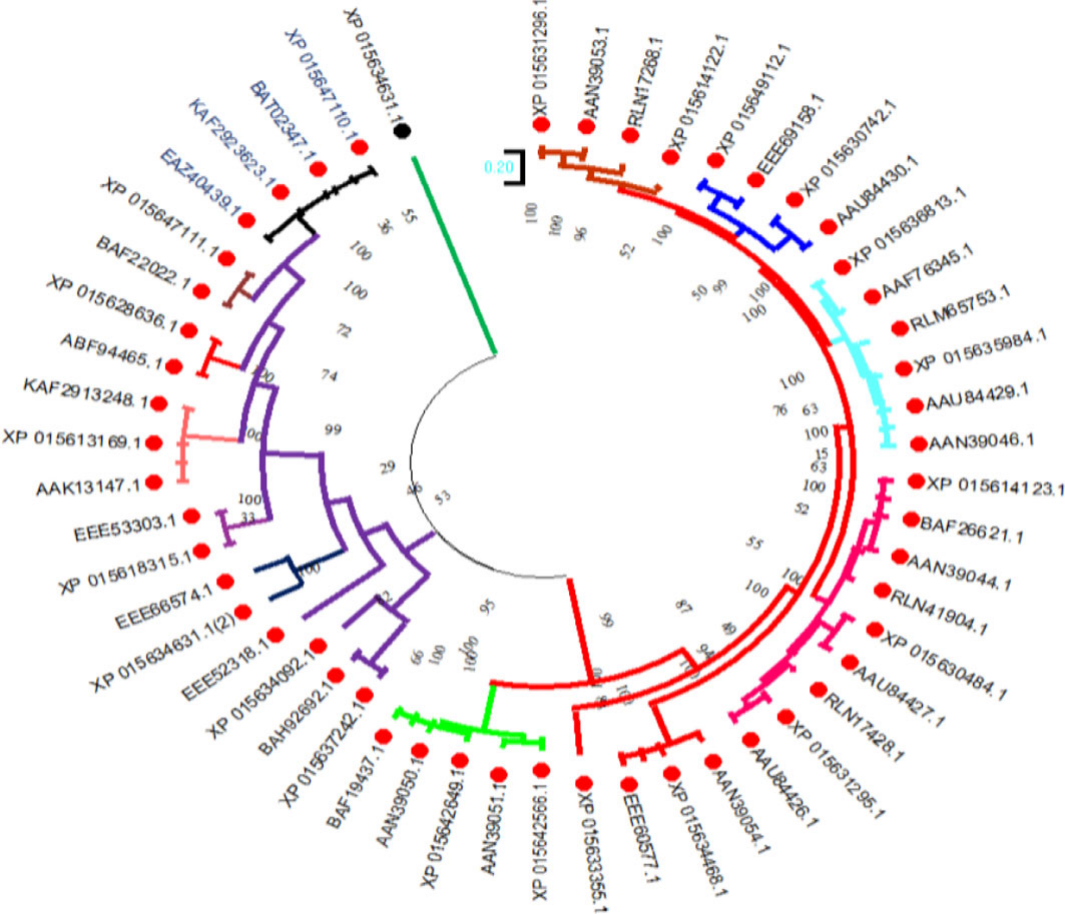
Phylogenetic analysis of selenite transporters among *Oryza sativa* L. selenite transporters. The trees were constructed by the maximum likelihood method with 500 bootstrap replicates.

To investigate the evolutionary relationships of the selenate transporters, amino acid sequences of proso millet selenate transporters and 32 selenate transporters sequences from *Arabidopsis thaliana* were used to construct the tree (**Figure 5**). In the same way, for constructing selenite transporter tree, we used proso millet selenite transporters and 52 selenite transporters from *Oryza sativa* L. (**Figure 6**). Tree branch lengths are measured in substitutions per site, with the tree drawn to scale. The proportion of sites where at least 1 unambiguous base is present in at least 1 sequence for each descendent clade is shown next to each internal node in the tree. Both the phylogenetic trees generated two main groups with many subgroups, indicating close relation and relative likeness between them.

The analysis of the promoter sequences of selenate transporters revealed that the promoter of RLM65282.1 is located 1000bp upstream, and for the RLN42222.1, RLN18407.1 and RLM74477.1. the locations of promoters were not visualized. Among selenite transporters, RLN41904.1 showed the promoter location above 200bp or 1000bp, RLN17428.1 showed promoter location upstream 500bp or 1000bp and for the RLN17268.1,RLM65753.1 the promoter location was above 1100bp.

The analysis of the promoter sequences of selenate transporters revealed that the promoter of RLM65282.1 is located 1000bp upstream, and for the RLN42222.1, RLN18407.1 and RLM74477.1. the locations of promoters were not visualized. Among selenite transporters, RLN41904.1 showed the promoter location above 200bp or 1000bp, RLN17428.1 showed promoter location upstream 500bp or 1000bp and for the RLN17268.1, RLM65753.1 the promoter location was above 1100bp.

### Protein-protein interaction analysis

**Figure 7:**
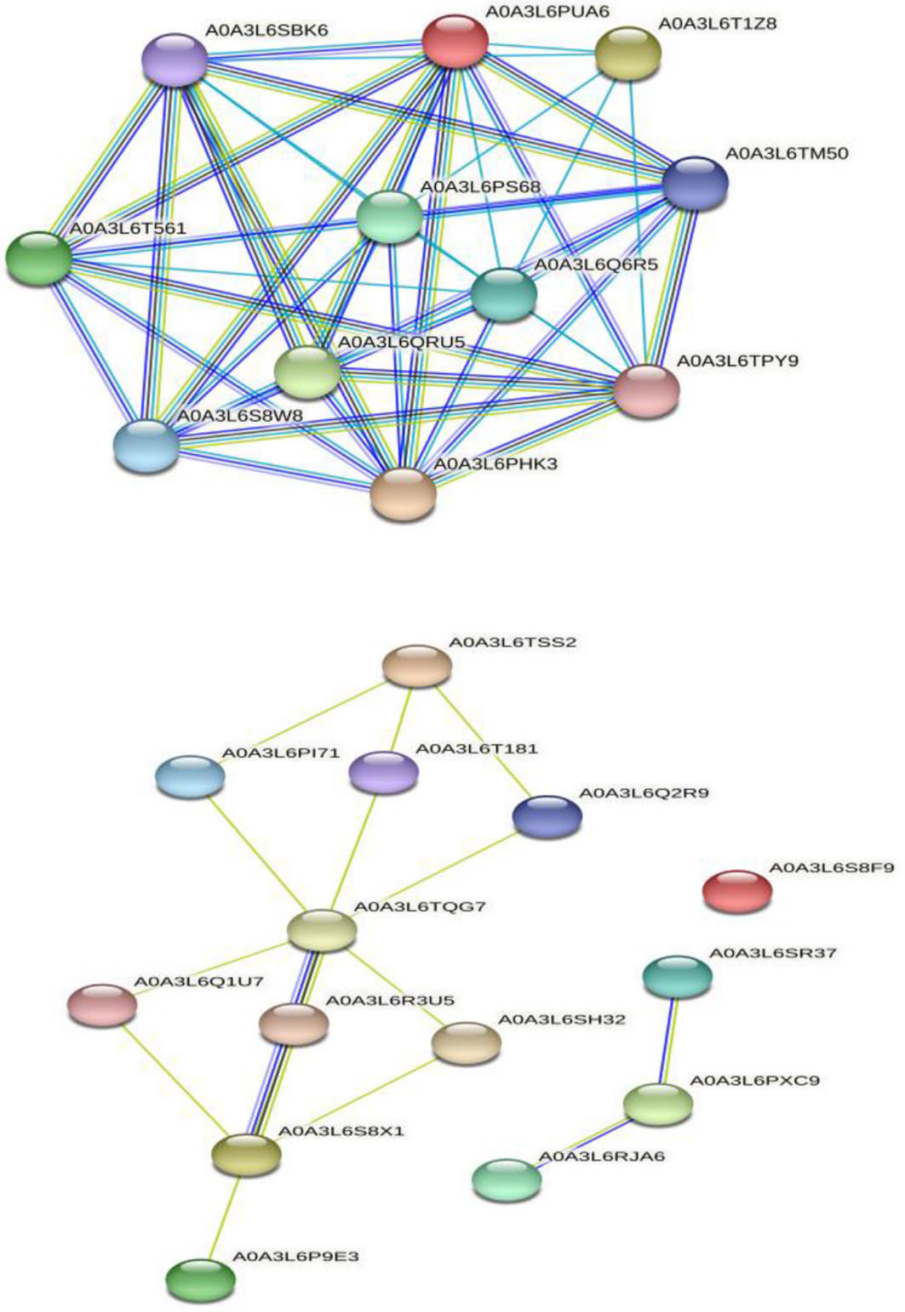
Protein-protein interaction analysis of selenate and selenite transporters, respectively.

The interaction among selected selenate (Nazir et al., 2021) and selenite (Nazir et al., 2021) transporters was done by multiple sequence searches with *Panicum miliaceum* L. revealing that 11 proteins are significantly involved in interaction with selenate transporters. However, the selenite transporters interacted with 10 proteins of *Panicum miliaceum* L., apart from this, three other selenite transporters showed interaction with each other. It is noteworthy that A0A3L6S8F9 do not appear to interact with any of the proteins. The PPI analysis is shown in **Figure 7**. Broadly, the selenate transporter proteins interact with sulfite oxidase like, adenylyl sulfate kinase and HIT domain containing proteins, whereas selenite transporters, interacts with Actin-7-like, H(+)/Pi cotransporter, rhamnogalacturonate lyase-like isoform X1, TPT, calcium uniporter protein and chloroplastic putative anion transporter 3.

### Three dimensional structure modeling and model assessment

**Figure 8:**
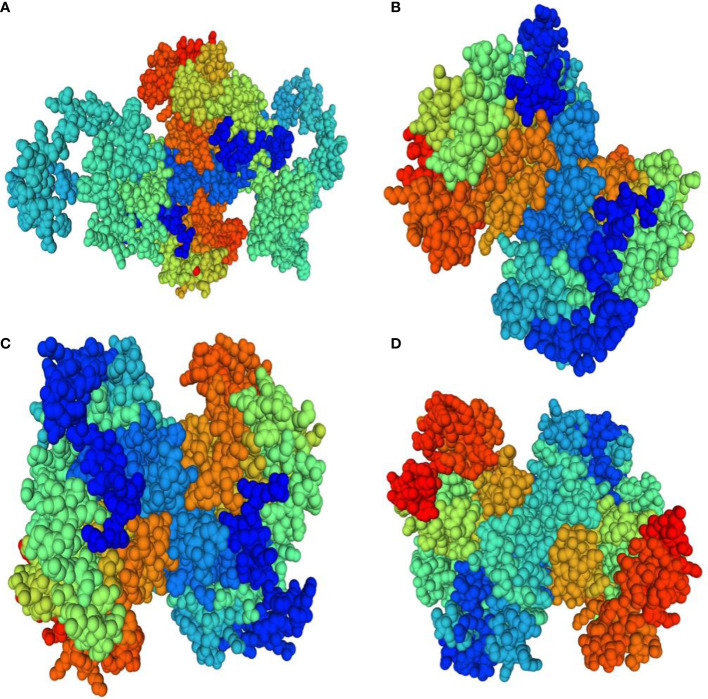
Predicted three dimensional structures of selenate transporters. **(A)** RLM65282.1 **(B)** RLN42222.1 **(C)** RLN18407.1 **(D)** RLM74477.1.

**Figure 9:**
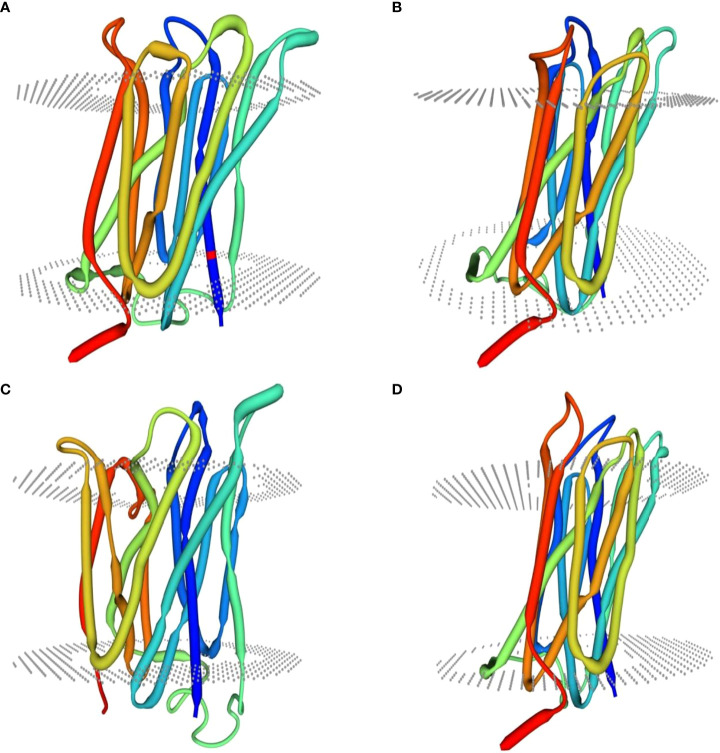
Predicted three dimensional structures of selenite transporters. **(A)** RLN41904.1 **(B)** RLN17428.1 **(C)** RLN17268.1 **(D)** RLM65753.1.

Homology modeling of selenate and selenite transporters was predicted using BLAST search. From the BLAST results, the selected template for all selenate transporters was revealed to be 4maf.1 (soybean ATP Sulfurylase) which showed an identity of 76.87%, 79.85%, 80.10% and 76.87% with RLM65282.1 (UniProt ID A0A3L6PUA6), RLN42222.1 (UniProt ID A0A3L6TPY9), RLN18407.1 (UniProt ID A0A3L6SBK6) and RLM74477.1 (UniProt ID A0A3L6QB84) respectively. The BLAST results for selenite transporters selected the template to be 7sp5.1 (eukaryotic phosphate transporter) which showed an identity of 34.9%, 34.61%, 40.09% and 34.54% with RLN41904.1 (UniProt ID A0A3L6TSS2), RLN17428.1 (UniProt ID A0A3L6S8X1), RLN17268.1 (UniProt ID A0A3L6S8F9) and RLM65753.1 (UniProt ID A0A3L6PXC9) respectively. The three-dimensional models selected for these transporters are shown in **Figures 8** and **9**. While assessing the structure of prepared models, it was found that A0A3L6PUA6, 82.23% (QMEAN -5.03) of amino acids were in Ramachandran’s favored region with no bad bonds. Similarly, the Ramachandran favored percentage for A0A3L6TPY9, A0A3L6SBK6 and A0A3L6QB84 was 95.43% (QMEAN 0.36), 96.65% (QMEAN 0.56) and 96.48% (QMEAN 0.21) with no bad angles receptively. In the same way the Ramachandran favored percentages for A0A3L6TSS2, A0A3L6S8X1, A0A3L6S8F9 and A0A3L6PXC9 were 92.14% (QMEAN -6.13), 91.95% (QMEAN -6.25), 91.02% (QMEAN -6.96) and 91.15% (QMEAN -6.31) respectively.

### Transcriptomic studies of identified transporters genes

To investigate the expression of selenate and selenite transporters under salt stress only and salt stress followed selenium. It was revealed that the expression of RLM65282.1 was 5.42 folds in control, 4.64 in salt-treated plants and 5.81 folds in plants treated with salt and selenium together. Similarly, the expression of RLN42222.1 and RLN18407.1 was 2.80, 2.76, 2.81 in control, salt and salt with selenium treated plants respectively. Among selenite transporter genes, the expression of inorganic phosphate transporter was 3.24, 2.46 and 3.95 folds in control, salt and salt with selenium treated plants respectively. The expression of putative transporters was found to be 1.7, 1.3 and 1.98 whereas for putative inorganic phosphate transporter it was 2.5, 2.5 and 2.6 respectively. The expression studies reveal that both selenate and selenite transporters are down-regulated with salt and up-regulated upon selenium applications.

## Discussion

In recent years, a major focus of plant researchers has been to understand the functions of membrane transporters in plants. Recent studies in *Oryza sativa* L. and *Arabidopsis thaliana* L. have revealed that phosphate and sulphate transporters play a vital role in Se uptake (Takahashi et al., 2000; Li et al., 2008; Barberon et al., 2008; Zhang et al., 2014), however, their role in millets remains relatively unclear and to date there is little data available on their identity and function in proso millet. In this study, eight Se transporters were identified and their expression was characterized. Among these, four (RLM65282.1, RLN42222.1, RLN18407.1 and RLM74477.1) belong to sulphate transporters which have a role in selenate uptake, whereas the other four (RLN41904.1, RLN17428.1, RLN17268.1 and RLM65753.1) function in selenite uptake. The role of sulphate transporters in selenate uptake and phosphate transporters in selenite uptake has been revealed in many studies (El Mehdawi et al., 2018; Wang et al., 2019; Li et al., 2020; Peng et al., 2021). Thirty-seven selenite transporters have been identified in *Pyrus malus*, 23 in *Camellia sinensis*, 42 in *populus* and 26 in rice (Liu et al., 2011; Zhang et al., 2016; Sun et al., 2017; Cao et al., 2021). Whereas, 12 selenate transporters have been identified in *Arabidopsis thaliana*, 11 each in *Populus trichocarpa* and *Sorghum bicolor* L., 12 in *Oryza sativa* L., 10 in *Triticum aestivum*, 8 in *Medicago truncatula*, eight in *Camellia sinensis* and 6 in *Astragalus racemosus* (Buchner et al., 2010; Zhang et al., 2022).

In this study, the physiochemical properties of selenate transporters revealed that the amino acid lengths of these transporters ranged from 474 to 646, whereas for selenite transporters it ranged from 521 to 539. The experimentally reviewed selenate transporter amino acid sequence of *Arabidopsis thaliana* L. (Q9LIK9) which was used as the source of BLAST has an amino acid length of 463 and the selenate transporter amino acids sequences of proso millet have more length than *Arabidopsis thaliana* L. Similarly the number of amino acid in selenite transporter sequence of *Oryza sativa* L. (Q8GSD9) is 528, indicating a similar number of amino acids with proso millet selenite transporters. RLM65282.1 has a higher molecular weight of 72171.69 Da among selenate transporters whereas among selenite transporters the highest molecular weight was 59138.29 Da which corresponds to RLM65753.1. The theoretical isoelectric point of selenate and selenite transporters ranged from 7.4 to 8.38 and 8.1 to 8.32 respectively. From these, it is clear that all these transporters are basic in nature (Moldoveanu and David, 2017). The unstable nature of protein is determined by the presence of certain dipeptides and is related to the protein’s half-life *in vivo*. The protein with an instability index smaller than 40 is predicted as stable and the value greater than 40 is predicted as unstable. Owing to these values all selenate transporters were found to be unstable, whereas all selenite transporters were stable (Guruprasad et al., 1990). A measure of the relative volume occupied by aliphatic amino acids is known as the aliphatic index which is directly correlated with the thermo stability of globular proteins (Ikai, 1980). The aliphatic index of selenate transporters ranges from 80.42 to 89.85 whereas that of selenite transporters ranges from 81.17 to 92.59, indicating higher stability of selenite transporters over a wide temperature range. Similarly, the negative grand average of the hydropathicity value indicates the hydrophilicity of the protein and vice versa (Baloji et al., 2019). The calculated grand average of hydropathicity for selenate transporters revealed that these are hydrophilic in nature, whereas, all selenite transporters are hydrophobic in nature, which infer that selenate proteins are more water soluble than selenite proteins which is corroborated with a study on tea plant (Cao et al., 2021). The predicted sub-cellular localization of RLM65282.1, RLN42222.1, RLN18407.1 and RLM74477.1 were mitochondria, chloroplast, chloroplast and mitochondria respectively, whereas all four selenite transporters are localized in plasma membrane. No trans-membrane helices for selenate transporters were found, whereas selenite transporters have 11 to 12 trans-membrane helices. Comparable results for sub-cellular localization of sulphate transporters and phosphate transporter were found in *Arabidopsis thaliana* (Rotte and Leustek, 2000; Araie et al., 2011; Młodzińska and Zboińska, 2016; Liao et al., 2019). The chromosomal locations of these transporters indicate that all selenate transporters (RLM65282.1, RLN42222.1, RLN18407.1 and RLM74477.1) are located on chromosome number 16, 1, 2 and 15. The selenite transporters (RLN41904.1, RLN17428.1, RLN17268.1 and RLM65753.1) are located on chromosome number 1, 2, 2 and 16. To investigate the evolutionary relationships of the Se transporters, amino acid sequences from proso millet and *Arabidopsis thaliana* L. were used to construct the tree. Similarly, for constructing a selenite transporter tree, sequences from proso millet and *Oryza sativa* L. were used. Both phylogenetic trees generated two main groups with many subgroups, indicating close relation and relative likeness between them. Our study correlated with similar studies in *Arabidopsis thaliana*, *Oryza sativa* L., *Camellia sinensis*, *Pyrus malus*, and *Populus trichocarpa*. A phylogenetic tree of phosphate transporter genes was constructed for *Arabidopsis thaliana*, *Oryza sativa* L., *Camellia sinensis*, *Pyrus malus*, and *Populus trichocarpa*. Using this information, genes were grouped into five clusters showing close relationships. Similarly, a phylogenetic tree based on *Arabidopsis thaliana*, *Oryza sativa*, *Populus tremula* x *alba*, and *Brassica oleracea* was constructed. Based on the phylogenetic tree, eight of the sulfate transporter genes clustered within the same branch as those found in *Populus tremula* × *alba* and they had closer genetic relationships from the perspective of gene evolution (Cao et al., 2021; Zhang et al., 2022). A protein/protein interaction study revealed that 11 proteins are significantly involved in interaction with selenate transporters and 10 proteins are involved with selenite transporters, apart from this, three other selenite transporters showed interaction with each other and one transporter (A0A3L6S8F9) did not appear to interact with any protein. The selenate proteins interact with sulfite oxidase-like (catalyzes the oxidation of sulfite to sulfate), adenylyl sulfate kinase (catalyzes the synthesis of activated sulfate) and HIT domain-containing proteins. Our study correlated with various previous studies in which they revealed similar interacting proteins with these transporters (Kopriva, 2006; Lewandowska and Sirko, 2008; Takahashi et al., 2011). Among selenite transporters, A0A3L6S8X1 interacts with Actin-7-like (required for the trafficking and endocytic recycling cytoplasmic streaming, cell shape determination and cell division), H(+)/Pi cotransporter (Integral component of membrane, inorganic phosphate transmembrane transporter activity, phosphate ion transport and symporter activity), rhamnogalacturonate lyase-like isoform X1 (carbohydrate binding and lyase activity), TPT domain-containing protein (integral component of membrane, organic anion transmembrane transporter activity and organophosphate ester transmembrane transporter activity) and an uncharacterized protein which is an essential component of the cell cytoskeleton, cytoplasmic streaming, cell shape determination, cell division, organelle movement and extension growth. A0A3L6PXC9 interacts with calcium uniporter protein and putative anion transporter 3, chloroplastic. The 3D structure assessment scores were calculated and models were validated by means of the Ramachandran plot. While assessing the structure of prepared models, it was found for A0A3L6PUA6, 82.23% (QMEAN -5.03) of amino acids were in Ramachandran favored region, for A0A3L6TPY9, A0A3L6SBK6 and A0A3L6QB84, 95.43% (QMEAN 0.36), 96.65% (QMEAN 0.56) and 96.48% (QMEAN 0.21) amino acids were in Ramachandran favored region with no bad angles receptively. In the same way the Ramachandran favored percentages for A0A3L6TSS2, A0A3L6S8X1, A0A3L6S8F9 and A0A3L6PXC9 were 92.14% (QMEAN -6.13), 91.95% (QMEAN -6.25), 91.02% (QMEAN -6.96) and 91.15% (QMEAN -6.31) respectively. This indicates the stability of all the models. QMEAN Z-scores around 0.0 reflect a native-like structure and, a QMEAN Z-score below 4.0 indicates a model with low quality (Benkert et al., 2011). Accordingly, A0A3L6TPY9, A0A3L6SBK6 and A0A3L6QB84 had native-like structures. The expression studies reveal that both selenate and selenite transporters are down-regulated with salt and up-regulated after applications of Se with salt. Se has a positive role in stress easing in plants (section 3.6). The up-regulation of selenate and selenite transporters under Se application can protect plants in various ways. Despite its non-essential nature, at lower doses Se has physiological benefits to plants, Se regulates the antioxidant system, enhances the chloroplast defense system, increases the ability of plants to scavenge excess ROS, promotes growth and photosynthesis, alleviates the damage to chloroplast ultrastructure, increase proline content, reduced H_2_O_2_ and MDA concentrations, enhanced biomass accumulation and relative water content, membrane stability index, stomatal conductance, photochemical efficiency and thus aiding them in mitigating stress (Tajima, 1993; Taha et al., 2021; Ahmad et al., 2021). In terms of the medicinal plant Iranian Borage, foliar application of sodium selenite resulted in higher levels of antioxidant activity and soluble sugars (Hosseinzadeh Rostam Kalaei et al., 2022), an effect that is very much similar to that on potato (Turakainen et al., 2004). As a consequence, the most frequent metabolic processes that follow the biosynthesis of carbohydrates exist in the pathway “Starch and sucrose metabolism” in which CAZymes acting through the double displacement catalytic mechanism are enriched towards the acquisition of energy required to cope with different biotic and abiotic stresses (Henrissat et al., 2001). Under salt stress, nano-selenium improved the antioxidant machinery and soluble sugars in groundnut (*Arachis hypogaea*). Studies on lettuce plants revealed the role of selenite and selenate in the alleviation of salt stress (Hawrylak-Nowak, 2015). Exogenous selenium in the form of selenate on olive reduced the negative effects of salt stress (Regni et al., 2021). In wheat, selenium and sulfur in involved in the mitigation of abiotic stress (Khan et al., 2015). Various similar studies revealed the role of selenium in abiotic stress mitigation (Hasanuzzaman et al., 2020; Shah et al., 2020; Mushtaq et al., 2021; Taha et al., 2021; Saleem et al., 2021; Rasool et al., 2022).

## Conclusion

Both phosphate and sulphate transporters in millets have been assessed using computational and expression studies. In the present study, it can be concluded that selenite may be transported through phosphate transporters and selenate through sulfate transporters. Expression studies revealed that both transporters play a vital role in salt stress mitigation in millets. This study also revealed that selenium may have a direct impact on the plant’s response to salt stress. The study of gene expression proved the possible role of selenate and selenite under salt and in turn selenium uptake. It would be beneficial to identify and study these transporters to better understand how the millets respond and tolerate salt stress. Future research in the field of stress physiology and agriculture is required for understanding selenium accumulation, its function, and its transport mechanism in plants. This study will provide the scientific basis and theoretical framework for future studies relating to selenium, millets, and salt stress.

## Data availability statement

The raw data supporting the conclusions of this article will be made available by the authors, without undue reservation.

## Author contributions

Conceptualization: NM, KA, SS, IT, AB, RR, and KH; data curation: NM, SS, RR, and KH; formal analysis: NM, FS, KA, SS, IT, AB, RR, and KH; funding acquisition: NM, SS, AB, RR, and KH; methodology: NM, KA, SS, FS, IT, AB, RR, and KH; software: AB, RR, and KH; validation: NM, KA, RR, and KH; visualization: RR and KH; writing – original draft: NM, KA, SS, IT, FS, AB, RR, and KH; writing – review and editing: KA, AB, RR, and KH. All authors contributed to the article and approved the submitted version.

## Funding

This research work was funded by Institutional Fund Projects under grant no (IFPRC-219-130-2020). Therefore, authors gratefully acknowledge technical and financial support from the Ministry of Education and King Abdulaziz University, Jeddah, Saudi Arabia.

## Conflict of interest

The authors declare that the research was conducted in the absence of any commercial or financial relationships that could be construed as a potential conflict of interest.

## Publisher’s note

All claims expressed in this article are solely those of the authors and do not necessarily represent those of their affiliated organizations, or those of the publisher, the editors and the reviewers. Any product that may be evaluated in this article, or claim that may be made by its manufacturer, is not guaranteed or endorsed by the publisher.
